# Novel targeted therapies for Parkinson’s disease

**DOI:** 10.1186/s10020-021-00279-2

**Published:** 2021-02-25

**Authors:** Theodora Ntetsika, Paraskevi-Evita Papathoma, Ioanna Markaki

**Affiliations:** 1 Department of Clinical Neuroscience, Karolinska Institutet, Stockholm, Sweden; 2 Center of Neurology, Academic Specialist Center, Solnavägen 1E, 113 65 Stockholm, Sweden; 3 Department of Neurology, Danderyd Hospital Stockholm, Stockholm, Sweden

**Keywords:** Parkinson’s disease, Therapeutics, Drug development

## Abstract

Parkinson’s disease (PD) is the second more common neurodegenerative disease with increasing incidence worldwide associated to the population ageing. Despite increasing awareness and significant research advancements, treatment options comprise dopamine repleting, symptomatic therapies that have significantly increased quality of life and life expectancy, but no therapies that halt or reverse disease progression, which remain a great, unmet goal in PD research. Large biomarker development programs are undertaken to identify disease signatures that will improve patient selection and outcome measures in clinical trials. In this review, we summarize PD-related mechanisms that can serve as targets of therapeutic interventions aiming to slow or modify disease progression, as well as previous and ongoing clinical trials in each field, and discuss future perspectives.

## Background

Parkinson’s disease (PD) is the second most common neurodegenerative disorder worldwide, affecting 2–3 % of the population ≥ 65 years of age (Pringsheim et al. 2014). Incidence estimates range from 5 to > 35 new cases per 100,000 individuals each year (Twelves et al. 2003) and while the figures are expected to further increase with the ageing of population (Marras et al. 2018), no disease modifying therapies are yet available (Espay et al. 2018). Levodopa brought a revolution in the field of management of PD by significantly improving parkinsonian symptoms, quality of life, and normalizing life expectancy (Jankovic 2002; Tambasco et al. 2018). More recent dopaminergic therapies, including dopamine agonists, monoamine oxidase B inhibitors, catechol-O-methyltransferase inhibitors (Rascol et al. 2003) as well as several unique formulations of levodopa, have been developed to address levodopa therapy shortcomings (Armstrong et al. 2020). Continuous duodenal infusion of levodopa/carbidopa intestinal gel and apomorphine subcutaneous pumps are such examples (Armstrong et al. 2020). Also, deep brain stimulation (DBS) is a very useful approach for patients with motor complications not responsive to medication adjustments (Armstrong et al. 2020). All these therapies have been of great value in the management of PD symptoms, although wearing out effect of oral medications, limited therapeutical options for patients with advanced disease, and the failure of clinical trials on disease modifying agents so far, have led researchers to reconsider clinical trial design (Cedarbaum 2018). Current and future efforts in the development of PD treatments involve the development and application of biomarkers that improve and objectify measurements of target engagement, pharmacokinetics and pharmacodynamics, disease state, safety and disease outcome (Cedarbaum 2018).

With regard to PD pathophysiology, the selective neuronal death in substantia nigra pars compacta constitutes a landmark finding, followed by the description of the classical basal ganglia model comprising the direct, indirect and the more recently added, hyperdirect pathways that regulate the control of movement (Albin et al. 1989; Kish et al. 1988), as well as the most recent view of PD as a systemic disorder, with profound involvement of the peripheral and enteric nervous system (Beach et al. 2010). The original PD model was mainly developed from data collected in rodents and primates with tract-tracing invasive approaches, immunochemistry and in situ hybridization techniques (Quartarone et al. 2020), and it correlates poorly with a considerable fraction of parkinsonian symptoms. Thus, a more systemic approach is necessary to describe pathological processes in PD as a multifactorial disease with complex symptom pattern (Obeso et al. 2017). Following the Braak theory of ascending pathology from the brainstem towards the cortex, in the PD brain (Braak et al. 2003), the research focus has moved towards the network degeneration hypothesis. Multimodal neuroimaging including functional magnetic resonance tomography (MRI), blood oxygen level-dependent (BOLD) MRI, and positron emission tomography (PET) have been utilized in connectivity studies. Despite the lack of reliable a-synuclein tracers, as opposed to histopathological studies, functional neuroimaging enables longitudinal monitoring of disease mechanisms along with symptom progression, thereby providing tools that increase diagnostic accuracy (Pagano et al. 2016; Politis 2014). A disease-specific metabolic pattern based on ^18^F-fluorodexyglucose (FDG)-PET has been developed and repeatedly validated in PD populations and has also been suggested as an objective tool of tracking treatment effect in clinical trials (Schindlbeck et al. 2018). In a recent, trimodal approach comprising ^18^F-FDG-PET, functional MRI and ^18^F-DOPA-PET, evidence was found for network-dependent neurodegeneration in PD by recapitulating the impact of nigrostriatal pathway impairment on putaminal dopamine depletion to striatocortical motor circuit dysfunction, thus providing a biomarker for the quantification of disease progression (Ruppert et al. 2020). In the present review, we summarize novel pharmacotherapeutic and non-pharmacological approaches with specific mention in clinical trials that apply neuroimaging and other objective disease-specific biomarkers on patient selection and treatment effect measurements.

### Gene therapies

Gene therapy is a rapid evolving, genome editing technology aiming to treat a disease by genetically modifying populations of cells that are either directly functionally impaired or capable of relieving disease symptoms (Coune et al. 2012). The technology is based on the use of a vector to carry DNA, RNA, antisense oligonucleotides or DNA- or RNA-editing enzymes into specific cells to modulate gene expression (Borel et al. 2014; Haggerty et al. 2020; Han et al. 2019; Hudry et al. 2019). Increasing clinical evidence of viral vector-based gene therapy approaches is available in PD (Axelsen et al. 2018; Fiandaca et al. 2010), as a result of studies on animal models that provided proof for the safety and efficacy of two families of viral vectors, characterized by both durable gene expression in neurons and minimal immunogenicity: adeno-associated viruses (AAVs) and lentiviruses (LVs)(Wong et al. 2006; Wu et al. 2006). AAVs have been widely used as vectors in central nervous system (CNS) disorders (Cearley et al. 2007). The AAV serotype 2 (AAV2) has demonstrated excellent tropism for neurons (Cearley et al. 2007; Fiandaca et al. 2009), while other AAV serotypes have been used for targeting other cell populations in the CNS, such as astrocytes (Hanlon et al. 2019) and microglia (Rosario et al. 2016). Also, AAV2 vectors are characterized by limited risk of insertional mutagenesis for the host (Berns et al. 2017; Gao et al. 2005) and effective expression after one-time delivery treatment (Christine et al. 2019).

The AAV serotype 9 (AAV9) has the highest tropism for the CNS (Kantor et al. 2014), and it is further advanced by the use of self-complementary vectors that significantly increase the viral transduction in several tissues (McCarty 2008), including the adult motor neurons in the spinal cord, due to its unique ability to penetrate the blood brain barrier (Duque et al. 2009). Based on these properties, AAV9 was investigated in mice models of spinal muscular atrophy (SMA), a monogenic disease characterized by the degeneration of the spinal motor neurons, with positive results in motor neuron survival, reversal of the phenotype and increased survival (Dominguez et al. 2011; Foust et al. 2010; Valori et al. 2010). Subsequent studies on non-human primates (Bevan et al. 2011; Dehay et al. 2012) and one clinical trial on patients with SMA (Mendell et al. 2017) confirmed the safety and efficacy of treatment, and Zolgensma^→^ was the first gene therapy to be approved in 2019 (Hoy 2019).

Lentivectors have been developed from primate lentiviruses, such as the wild-type human immunodeficiency virus type 1 and non-primate lentiviruses, such as the equine infectious anemia virus, by progressively removing most of the viral genes from the vector genome to limit the risk of producing replication-competent viral particles (Cronin et al. 2005; Dull et al. 1998; Naldini et al. 1996; Olsen 1998; Zufferey et al. 1998; Zufferey et al. 1997). Lentivectors tropism can be specifically modified through pseudotyping strategies (Naldini et al. 1996). However, LVs can increase the risk for insertional mutagenesis, due to their capacity to integrate into the host genome (Coune et al. 2012), and several strategies have been introduced to improve their safety profile, including directing integration to heterochromatin regions of the genome, introduction of self-inactivating mutations, production of non-integrating lentivectors and, more recently, removal of a sequence element involved in plus-strand DNA synthesis shown to further reduce integration and increase the efficiency of formation of circular episomes (Apolonia et al. 2007; Gijsbers et al. 2010; Kantor et al. 2011; Philippe et al. 2006; Zufferey et al. 1998).

Gene therapy clinical trials in PD have focused on 4 main targeted approaches: (1) restoring dopamine synthesis, (2) neuroprotection, (3) genetic neuromodulation and (4) addressing disease-specific pathogenic variants (Merola et al. 2020). Gene therapies targeting pathogenic GBA variants are addressed in the section “Glucocerebrosidase targeting therapies”.

PD trials focusing on dopamine restoration strategies have targeted either aromatic L-amino acid decarboxylase (AADC) alone using AAV2 as vectors (AAV2-AADC) (Christine et al. 2019; Christine et al. 2009; Mittermeyer et al. 2012; Muramatsu et al. 2010) or a triad of key enzymes in the dopamine biosynthetic pathway including AADC, tyrosine hydroxylase (TH) and GTP-cyclohydrolase (GCH1) using lentivectors (LV-GCH1-TH-AADC; ProSavin^→^) (Palfi et al. 2014). AAV-AADC phase-I clinical trials demonstrated safety and a significant improvement of both motor and non-motor symptoms as assessed by the Unified Parkinson Disease Rating Scale (UPDRS), a decrease in OFF-time duration without an increased effect of ON-time dyskinesias, as well as an increase in the uptake of the AADC tracer at PET, which was used as a measure of gene expression (Christine et al. 2009; Mittermeyer et al. 2012; Muramatsu et al. 2010). A phase-Ib study demonstrated a dose-dependent improvement of clinical outcomes, including increase in ON-time duration without dyskinesias and quality of life, dopaminergic medications reduction, and AADC enzymatic activity assessed by PET (Christine et al. 2019). These results led to an ongoing phase-II, randomized, sham surgery controlled, double-blind, multi-center clinical trial (NCT03562494, Table 1) to primarily assess changes in ON-time duration without dyskinesias, as recorded by participants in their PD diary. As for LV-GCH1-TH-AADC, results of a phase I/II trial with two study sites (France and UK) showed long-term safety and tolerability of ProSavin^Ⓡ^, as well motor symptom improvement, as assessed with the UPDRS part 3 motor score. An improved version of ProSavin^Ⓡ^, OXB-102 (NCT03720418, Table 1), is under investigation in a two-phase trial including an open-label, dose-finding phase, in which patients will receive one of three escalating doses, and a randomized, double-blind phase in which patients will be randomized to either an active group receiving the selected dose from phase 1, or a control group.

**Table 1:** Current non-pharmaceutical disease-modifying ongoing clinical trials

Mechanism	Intervention	ClinTrial Indentifier	Phase	Τarget population	Primary outcome	Other secondary outcomes of interest	Sponsor
Gene therapies	Vector genome: VY-AADC02	NCT03562494	II	Moderate to Advanced PD with motor Fluctuations	Changes in ON time without troublesome dyskinesia	.	Neurocrine Biosciences
	Lentiviral vector: OXB-102	NCT03720418	I/II	Bilateral PD	AE, SAE, Changes in MRI	.	Sio Gene Therapies
Neuromodulation	MRgFUS	NCT04002596 NCT03319485 NCT02263885 NCT02246374 NCT03608553	NA	Advanced PD	AE, Responders, Changes in CRST, Changes in MDS-UPDRS	.	InSightec
Neuromodulation	rTMS	NCT04238000	NA	PD	Motor Outcomes	.	Fondazione Europea di Ricerca Biomedica Ferb Onlus
		NCT04431570	NA	PD, freezing of gait	Changes in FOGT	fMRI	Peking Union Medical College Hospital
		NCT04116216	II	PD	Changes in MDS-UPDRS part III	.	Universidade Federal de Pernambuco
		NCT03836950	I/II	PD	Changes in Cognitive Outcomes	.	VA Office of Research and Development
		NCT02346708	NA	PD with MCI	Changes in magnetoencephalography connectivity measures	.	University of Colorado, Denver
		NCT03552861	NA	PD with Depression or Cognitive Impairment	Changes in HAMD and BDI	.	Guangdong Provincial People’s Hospital

*AE* Adverse Events, *BDI* Beck Depression Inventory, *CRST* Clinical Rating Scale for Tremor, *fMRI *functional Magnetic Resonance Imaging, *FOGT* Freezing Of Gait Questionnaire, *HAMD* Hamilton Depression Scale, *MCI* Mild Cognitive Impairment, *MDS-UPDRS* MDS-Unified Parkinson’s Disease Rating Scale, *MRgFUS* Magnetic Resonance Guided Focused Ultrasound, *MRI* Magnetic Resonance Imaging, *NA* Not Applicable; *rTMS* Repetitive Transcranial Magnetic Stimulation, *PD *Parkinson’s Disease

**Fig. 1:**
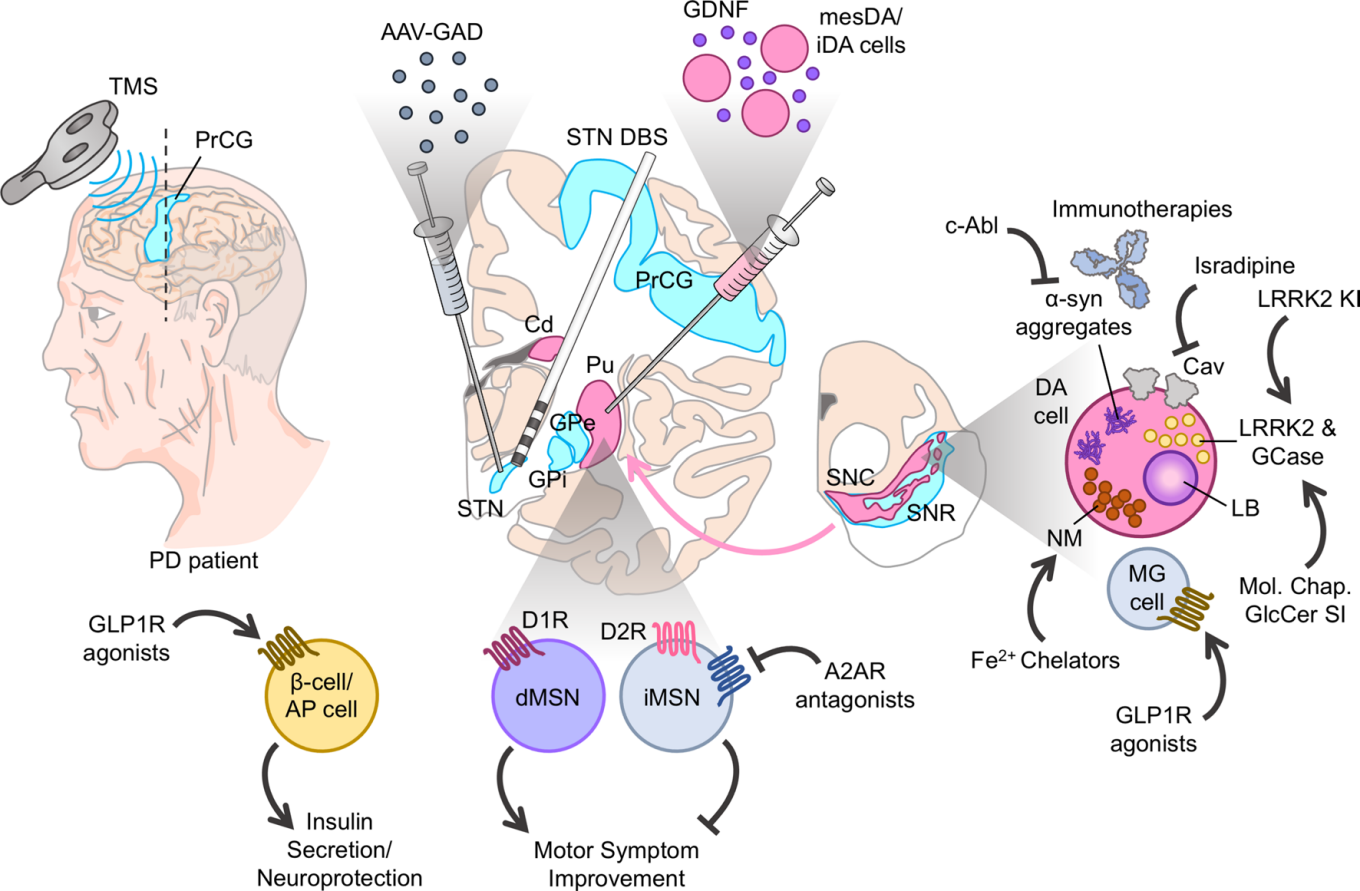
Potential neuroprotective mechanisms of novel targeted therapies in Parkinson’s disease. *AAV* adeno-associated virus, *AP* area postrema, *A2AR* adenosine A2A receptor, *Cd* caudate nucleus, *DA cell* dopaminergic cell, *DBS* deep brain stimulation, *dMSN* direct pathway medium spiny neurons, *D1R* dopamine D_1_ receptor; *D2R* dopamine D_2_ receptor; *GAD* glutamate decarboxylase; *GCase* glucocerebrosidase, *GDNF* glial cell-line derived neurotrophic factor, *GLP1R* glucagon-like peptide 1 receptor, *GPe* globus pallidus externa, *GPi* globus pallidus interna, *iMSN* indirect pathway medium spiny neurons; *KI* kinase inhibitor, *LB* lewy body, *LRRK2* leukine-rich repeat kinase 2, *MG* microglia, *NM* neuromelanin; *PrCG* pre-central gyus, *Pu* putamen; *SNC* substantia nigra pars compacta, *SNR* substantia nigra pars reticulata, *STN* subthalamic nucleus, *TMS* transcranial magnetic stimulation

Two neurotrophic factor gene therapies delivered via AAV2 vectors have provided either neurturin (AAV2-NRTN) or glial cell-line derived neurotrophic factor (AAV2-GDNF) to PD patients. GDNF is a 134 amino acid protein belonging in the GDNF family ligands and the most potent trophic factor of dopaminergic neurons (Lin et al. 1993). After years of studies in preclinical models including GDNF-transgene infusion as well as direct GDNF protein infusion intraparenchymally or intraventricularly (Gash et al. 1996; Kells et al. 2010; Pascual et al. 2011; Richardson et al. 2011; Tomac et al. 1995), several clinical trials have been developed to investigate the efficacy of GDNF treatment in the management of PD. In a phase 1 safety trial, GDNF delivered directly into patients’ putamen (Fig. 1) showed no serious clinical side effects, improvement of UPDRS part 2 and 3 scores, reduction of dyskinesias and significant increase in putamen dopamine storage assessed by PET (Gill et al. 2003). An escalation, dose-ranging trial using MRI monitored AAV2-GDNF putamen infusion showed good safety and tolerability of the therapy, substantial stability of the UPDRS scores up to 18 months post treatment and an increase in ^18^F-DOPA uptake 6 to 18 months post treatment, suggesting a neurotrophic effect on dopaminergic neurons (Heiss et al. 2019). GDNF administration using intermittent intraputaminal convection-enhanced delivery via a skull-mounted transcutaneous port showed less promising results (Whone et al. 2019). However, the patients that responded with an improvement of motor outcome as assessed by UPDRS part 3 continued to experience clinical improvement in an open-label extension study (Whone et al. 2019). An ongoing phase Ib trial (NCT04167540) is designed to evaluate the safety and potential clinical effect of MRI-guided intraputaminal delivery of AAV2-GDNF in two cohorts of patients with either early-stage or advanced PD, thus contributing with important knowledge on the therapeutic effect early in PD course.

Cerebral dopamine neurotrophic factor (CDNF) is an additional protein, 161 amino acids long, with structurally and functionally distinct properties compared to other neurotrophic factors (Lindahl et al. 2017), including the regulation of apoptosis and unfolded protein response, and reduction of glial cell secretion of proinflammatory cytokines. CDNF targets preferably injured cells and diffuses broadly within the tissues – a property that may result in benefits beyond the nigrostriatal pathway and motor symptom alleviation. A phase 1 clinical trial aiming to investigate the safety and tolerability of intraputaminal infusion of CDNF in patients with advanced PD and motor fluctuations (NCT03295786, NCT03775538) is recently completed and a long-term follow-up, safety study (NCT04228653) is ongoing until March 2023. CDNF is delivered intracerebrally with an implanted drug delivery system, thereby not being a gene therapy, yet included in this section of the Review, along with other neurotrophic factors.

Neurturin, a structurally 42 % similar to GDNF neurotrophic factor expressed in the substantia nigra and striatum (Kotzbauer et al. 1996), has been another target for AAV2 mediated gene therapy (CERE-120). A phase I dose-escalating, open-label study designed to assess the safety, tolerability and biologic activity of CERE-120 revealed no clinically significant side effects and improvement of UPDRS motor score both in ON and OFF-medication state, without troublesome dyskinesias one-year after treatment (Marks et al. 2008). However, a subsequent multi-center, double-blind, sham-surgery controlled trial showed no superiority of CERE-120 treatment to sham surgery, as assessed with the UPDRS motor score, during equally long follow-up period. Extended follow-up over 15 to 24 months also failed to demonstrate significant clinical improvement in CERE-120 group compared to the control group (Warren Olanow et al. 2015).

In order to restore inhibitory control of the subthalamic nucleus (STN) in patients with PD (Jahanshahi et al. 2015), gene therapy using AAV vectors to deliver glutamate decarboxylase (GAD) into the STN has been introduced (Fig. 1). GAD, the enzyme that catalyzes the formation of the inhibitory neurotransmitter gamma-aminobutyric acid (GABA), has two forms GAD65 and GAD67, expressed by two different genes (Erlander et al. 1991). The expression and activity of GADs is directly connected to GABA levels and subsequent GABAergic neurotransmission at the inhibitory synapse (Lee et al. 2019). An open label, phase I trial showed safety and tolerability of unilateral, subthalamic AAV-GAD injection, as well as significant improvements in the UPDRS motor score, predominantly on the side of the body that was contralateral to surgery up to 12 months after treatment (Kaplitt et al. 2007). These results prompted a phase-II, double-blind, sham-surgery controlled, randomized trial which showed a significantly greater improvement of the UPDRS sub-scores in the AAV-GAD group compared to the sham group, over a 6-month follow-up period (LeWitt et al. 2011). Long-term follow-up confirmed persistent clinical benefit, and good safety and tolerability outcome 12 months post treatment (Niethammer et al. 2017). FDG-PET scans performed preoperatively and at 6 and 12 months after surgery revealed the development of a unique, treatment-dependent, polysynaptic brain circuit linking the STN to motor cortical regions, which correlated with clinical improvement in the AAV2-GAD treated patients (Niethammer et al. 2018).

Thus, in clinical trials of gene therapies, outcome measures comprise both clinical improvement and functional neuroimaging markers of pharmacodynamics (e.g. dopaminergic neuron density and AADC activity). Also, in an attempt to closer explore drug effect on brain connectivity, consecutive FDG-PET scans were successfully combined with network analysis to provide insight in the metabolic signature of gene therapy, suggesting a new tool for the evaluation of therapeutic efficacy in PD.

### Targeting alpha‐synuclein

Alpha-synuclein (α-syn) is a 140-amino-acid protein encoded by the *SNCA* gene, that is abundant in the brain, and more specifically at the neuronal presynaptic terminals. Its function is not fully understood, but it seems to have a role in synaptic vesicle recycling and neurotransmitter release (Sulzer et al. 2019). Aggregation of α-syn and accumulation in cytoplasmic inclusions known as Lewy bodies, is the pathological hallmark of PD (Spillantini et al. 1997). Although the precise mechanism of toxicity is still to be revealed, current disease-modifying therapies focus on targeting the spread, production, aggregation, and degradation of α-syn.

#### Decreasing the expression of a-synuclein

Manipulation of α-syn levels by gene silencing with RNA interference has been shown to be beneficial in normalizing α-syn expression and improve motor function in experimental studies (McCormack et al. 2010; Takahashi et al. 2015), yet fine-tuned balance is necessary to avoid nigrostriatal neurotoxicity caused by excess downregulation (Gorbatyuk et al. 2010). DNA methylation at SNCA intron 1 is a regulator of the α-syn transcription, and methylation levels differ in PD compared to controls (Jowaed et al. 2010), thus providing a target for tight control of α-syn expression levels. Novel clustered regularly interspaced short palindromic repeats technology has been successfully used in fine tuning the downregulation of SNCA expression in stem cell-derived dopaminergic neurons, suggesting a new approach (Kantor et al. 2018).

#### Prevention of α-syn aggregation

High-throughput screening of compound libraries in combination with medicinal chemistry optimization have recently led to the development of the novel oligomer modulator anle138b, which showed to inhibit the formation of pathological oligomers in-vitro, as well as in several mouse models of prion and PD (Wagner et al. 2013). The first in human clinical trial on healthy volunteers was completed in august 2020 (NCT04208152), and reported no side effects in doses up to 300 mg. Also, plasma levels exceeded those required for efficacy in animal models, and the uptake was not affected by food. Based on these results, further funding was secured for testing in PD patients.

Heat shock proteins (HSPs) are small molecular chaperones that serve in protein homeostasis and prevent protein aggregation and toxicity in conditions of cellular stress. Several HSPs have been observed as components of Lewy body inclusions, and manipulation of their expression in in-vitro and in-vivo models has been shown to modulate α-syn aggregation and toxicity (Sinnige et al. 2020), alluding that restoration of physiological proteostasis could serve as a therapeutic target in neurodegenerative diseases. Several other small molecules have been investigated in pre-clinical studies for their efficacy in preventing α-syn aggregation. Leuco-methylthioninium bis (hydromethanesulfonate) is one such compound that has been reported to prevent tau aggregation and has subsequently been tested in cell lines and a transgenic mouse model of PD with encouraging results (Schwab et al. 2017). NPT-100-18A is a de novo compound developed by Wrasidlo and colleagues through molecular methods that targeted the C-terminal of α-syn, which has an important role in dimerization and membrane penetration (Wrasidlo et al. 2016). As a cyclic peptidomimetic compound, NPT-100-18A interferes at the sites of α-syn monomer interaction, thus preventing oligomerization and toxicity. The results in in-vitro studies and in two different transgenic rodent models showed decreased a-syn aggregation, reduced cortical synaptic accumulation and normalization of inflammatory and neuronal markers (Wrasidlo et al. 2016). The molecular tweezer CLR01 inhibits α-syn aggregation through binding to lysine residues of α-syn that are crucial for its oligomerization, and it has shown efficacy, in terms of motor symptom improvement and decreased oligomeric α-syn burden, in transgenic mouse models of PD (Bengoa-Vergniory et al. 2020).

Nilotinib is a tyrosine kinase c-Abl inhibitor approved for the treatment of leukemia, that has also shown to increase autophagy and degradation of intracellular α-syn aggregates (Fig. 1). Phase 2 clinical trials in PD have recently concluded contradicting results regarding CNS bioavailability and alteration of dopamine metabolites, although the drug was safe and well tolerated in both studies (Simuni et al. 2020; Pagan et al. 2020). Critique has been addressed on the interpretation of the results of the published study (Pagan et al. 2020) regarding safety that has been questioned, as increasing frequency of serious adverse events was observed with increasing dose of nilotinib, and also regarding the effect of treatment on altering relevant biomarkers, as those were only measured at the end of treatment, with no baseline reference (Espay et al. 2020). Also, although the study was not an efficacy trial, it was concerning that patients in the nilotinob high-dose group showed deterioration in the activities of daily living, and the “Time Up and Go” motor test, as well as in the Montreal Cognitive Assessment score, at the end of treatment compared to baseline. Thus, a closer reflection on patient selection of potential responders, and on outcome measures that will reflect the targeted mechanism was suggested. Yet, another phase II clinical trial focusing on c-Abl inhibition (NCT03655236, Table 2) is ongoing, evaluating both clinical outcome and imaging-based biomarkers.

**Table 2:** Current pharmaceutical disease-modifying clinical trials phase II and III

Τargeting mechanism	ClinTrial indentifier	Drug	Phase	Τarget population	Primary outcome	Other secondary outcomes of interest	Sponsor
A2Α receptor antagonists	NCT03703570	KW-6356	II	PD	Changes in MDS-UPDRS part III	.	Kyowa Kirin Co., Ltd.
	NCT02939391	KW-6356	II	Early PD	Changes in MDS-UPDRS part III	.	Kyowa Kirin Co., Ltd.
Calcium Targeting Therapies	NCT02168842	Isradipine	III	Early PD	Changes in MDS UPDRS Part I-III	.	University of Rochester
Glucagon-like peptide 1 receptor agonists and other antidiabetic agents	NCT03659682	Semaglutide	II	Early PD	Changes in MDS-UPDRS part 3 in OFF medication state	.	Oslo University Hospital
	NCT02953665	Liraglutide	II	Early PD	Motor Function, Non-Motor Function, Cognitive Function	.	Cedars-Sinai Medical Center
	NCT03439943	Lixisenatide	II	Early PD	Changes in MDS-UPDRS Part III in ON status	.	University Hospital, Toulouse
	NCT04305002	Εxenatide	II	Early PD	Changes in FDG-PET network analysis	Changes in MDS-UPDRS Part III in ON and OFF status	Center for Neurology, Stockholm Karolinska Institutet
	NCT04154072	NLY01	ΙΙ	Early Treatment Naïve PD	Changes in MDS UPDRS Part II-III	.	Neuraly, Inc
	NCT04232969	Εxenatide	III	PD	Changes in MDS-UPDRS part III in OFF medication state	.	University College, London
	NCT04269642	Εxenatide	II	Early PD	Changes in MDS-UPDRS part III score	Changes in SNBR confirmed by PET scan	Peptron, Inc.
Glucocerebrosidase targeting therapies	NCT02914366	Αmbroxol	II	PD Dementia	Changes in ADAS-cog and ADCS-CGIC scales	Changes in MRI biomarkers	Lawson Health Research Institute
	NCT02906020	GZ/SAR402671	II	Patients with Early PD Carrying a GBA Gene Mutation	Number of Patients with AE, Changes in UPDRS Part II and III during OFF state	.	Genzyme, a Sanofi Company
	NCT04127578	PR001A	I/II	Patients with PD With at Least One GBA1 Mutation	Number of TEAEs and SAEs	.	Prevail Therapeutics
Targeting α-synuclein: Immunotherapies	NCT03100149	RO7046015/ PRX002	II	Early PD	Changes in MDS-UPDRS total score	Changes in DaT-SPECT	Hoffmann-La Roche
	NCT03318523	BIIB054	II	PD	Changes in MDS-UPDRS total score	Change in SBR measured by SPECT/DATSCAN	Biogen
Targeting α-synuclein: prevention of aggregation	NCT03655236	K0706	II	Early PD	Changes in MDS UPDRS Part II-III	Changes in DaT-SPECT	Sun Pharma Advanced Research Company Limited
Ιron Targeting Therapies	NCT02655315	Deferiprone	II	Treatment Naive PD	Changes in total MDS-UPDRS	.	University Hospital, Lille European Commission ApoPharma

*ADAS-cog* Alzheimer’s Disease Assessment Scale-cognitive subscale, *ADCS-CGIS* ADCS-Clinician’s Global Impression of Change, *AE* Adverse Events, *DATSCAN* Dopamine Transporter With Ioflupane I123, *FDG-PET* Positron emission tomography with 2-deoxy-2-[fluorine-18]fluoro- D-glucose, *MDS-UPDRS* Unified Parkinson’s Disease Rating Scale, *MRI *Magnetic Resonance Imaging, *PD* Parkinson’s Disease, *PET* positron emission tomography, *SAEs* Serious Adverse Events, *SNBR* specific to non-specific binding ratio, *SBR* Striatal Binding Ratio, *SPECT* Single Photon Emission Computed Tomography, *TEAEs* Treatment-Emergent Adverse Event

#### Immunotherapies targeting α-syn

Active immunization approaches encompass efforts to develop vaccines targeting the N or C-terminal of α-syn or its aggregation forms. Early clinical trials (NCT01568099, NCT01885494, NCT02216188, NCT02618941, NCT02267434) of the α-syn mimicking peptides PD01A (Volc et al. 2020) and PD03A in PD patients and healthy controls have shown good safety and tolerability, as well as sustainable immunogenicity over time, and phase 2 trials are under way to test clinical efficacy.

Passive immunization is based on the hypothesis that chronic intravenous administration of antibodies will halt the formation and spreading of pathogenic α-syn aggregates, and potentially modify the disease course. One phase 1 study has been completed (Jankovic et al. 2018) and several are ongoing (NCT03716570, NCT03272165, NCT03611569, NCT04127695); however, a major issue has been that only 0.1–0.2 % of the antibodies reach the CNS (Pardridge 2019), and that they act against the extracellular spreading but are unable to penetrate the cells (Gaston et al. 2019). Finally, it remains unclear which molecular species of α-syn is responsible for PD pathology and how (or if) it differs from those that cause other synucleinopathies. Phase 2 trials have included striatal binding of dopamine transporter in the secondary outcome measures (NCT03100149, NCT03318523, Table 2), which would be of interest to correlate with the clinical outcome measures, and the biofluid-measured alterations to improve understanding of the drug action mechanism. Preliminary results of the first part of the PASADENA study (NCT03100149) showed that it did not meet the primary objective (i.e. improvement of MDS-UPDRS total score by 37.5 % at 52 weeks), but patients in the prasinezumab group had significantly slower motor and non-motor symptom progression and improvement in imaging biomarkers consistent with disease modifying effect (Prothena Corporation 2020). Final results from the second part of the study, where the placebo arm will be re-randomized to one of the two prasinezumab doses and continue for another 52 weeks, will be of great interest and shed more light on the efficacy, mechanism of action and safety profile of the treatment.

### Glucocerebrosidase targeting therapies

Glucocerebrosidase (GCase) is a 497 amino-acid lysosomal hydroxylase, which degrades glucocerebroside into ceramide and glucose (Boer et al. 2020). Individuals that are homogenous for pathogenic variants of GBA, the gene encoding GCase, develop Gaucher’s disease due to excessive storage of glucocerebroside in the liver, spleen, bone, and bone marrow (Beutler 2001). Individuals who are heterozygous for GBA pathogenic variants though have an increased risk of parkinsonism and dementia (Tayebi et al. 2003), while GBA pathogenic variants are the most common known genetic cause of PD (Gan-Or et al. 2018). Also, patients with PD carrying GBA pathogenic variants have earlier age of disease onset, more rapid progression and reduced survival compared to patients without GBA pathogenic variants (Brockmann et al. 2015). GCase deficiency leads to accumulation of glucocerebroside in neurons that successively provokes formulation of toxic oligomers and decline in lysosomal proteolysis that preferentially affects α-syn. Conversely, elevated α-syn inhibits intracellular trafficking and lysosomal function of normal GCase in neurons, indicating the presence of a bidirectional pathologic loop between GCase and α-syn in PD and other synucleinopathies (Aflaki et al. 2017; Choi et al. 2011; Mazzulli et al. 2011; Sardi et al. 2015). Supported by these findings, many therapeutical strategies focusing on GCase have been introduced in recent years.

Small molecular chaperones that are able to cross the blood-brain barrier and increase the activity of lysosomal GCase in neurons has been one of these novel strategies (Fig. 1). These chaperones can bind to the pathologic enzyme in the endoplasmic reticulum (ER) enabling it to fulfill quality control requirements for lysosomal trafficking. Consequently, trafficking of the enzyme to the lysosome can decrease ER-associated degradation and increase lysosomal function (Bendikov-Bar et al. 2013; Lieberman et al. 2009). Treatment with the molecular chaperone ambroxol hydrochloride was found to improve lysosomal activity in fibroblast lines generated from skin biopsies of PD patients (McNeill et al. 2014), which led to an open label uncontrolled clinical trial. Eighteen patients with moderate PD received treatment with ambroxol for 186 days, which resulted in a significant increase in their CSF α-syn and GCase concentration levels as well as a significant decrease in UPDRS motor score, while safety and tolerability of the treatment was confirmed (Mullin et al. 2020). An ongoing, placebo-controlled, clinical trial is going to examine whether ambroxol therapy is associated with improvement of cognitive and motor symptoms of PD dementia (NCT02914366, Table 2). LTI-291 is another small-molecular activator of GCase (Alzforum 2020) currently being tested in GBA-associated PD (NTR6598, NTR6705, NTR6960, NTR7299). AT3375 is a next-generation GBA chaperone that has been suggested as a potential treatment both in Gaucher’s and PD (Khanna 2012).

Glucosylceramide synthase inhibitors have been shown to reduce the levels of glucosylceramide and glucosylsphingosine in the central nervous system, decelerate the accumulation of α-syn, ubiquitin and tau proteins and improve cognitive and behavioral outcome in a GBA-associated PD mouse model (Sardi et al. 2017). Subsequently, MOVES-PD global study was undertaken in order to assess safety, tolerability and efficacy of venglustat, a brain-penetrant allosteric glucosylceramide synthase inhibitor known also as GZ/SAR402671. So far, 270 participants, i.e. GBA mutation carriers with PD, are recruited and the study is estimated to be completed in 2024 (NCT02906020, Table 2).

GBA has also been one of the targets in AAV-mediated gene therapies. Preclinical models have shown the efficacy of AAV5-GBA and AAV9-GBA in preventing dopamine neuron loss (Rocha et al. 2015) and counteracting the widespread accumulation of α-syn deposits throughout the forebrain of transgenic mouse models (Morabito et al. 2017). Based on these data, an ongoing phase 1/2a, multicenter, open-label, ascending dose, first in-human clinical trial is planned to evaluate the safety of intracisternal PR001A (AAV9-GBA1) administration in patients with moderate to severe PD with at least one GBA pathogenic variant (NCT04127578, Table 2).

### LRRK2 targeting therapies

Leukine-rich repeat kinase 2 (LRRK2) is a member of the Ras-of-complex (ROC) protein family (West 2017). Pathogenic variants of LRRK2 gene are an important, relatively common cause of autosomal dominant PD (Funayama et al. 2002; Paisán-Ruíz et al. 2004; Zimprich et al. 2004), especially in particular ethnic groups (Kett et al. 2012). They are also observed in patients with sporadic PD, typically manifesting as late-onset PD closely resembling idiopathic PD in terms of clinical features and response to levodopa (Tolosa et al. 2020). The Gly2019Ser, the most common among LRRK2 pathogenic variants, localizing in the kinase domain of the protein, accounts for 4 % of familial and 1 % of sporadic PD worldwide (Tolosa et al. 2020). Genetic and biochemical data have shown that LRRK2 pathogenic variants — particularly Gly2019Ser and variants in the GTPase Roc and COR domains of the protein — cause a toxic, gain-of-function-mechanism mediated hyperactivity of LRRK2 kinase (Chan et al. 2017; Chen et al. 2018; Cookson 2017; Cresto et al. 2019; Healy et al. 2008; West 2017). Supported by these data, novel therapeutic approaches for LRRK2-associated PD, as well as idiopathic PD, have focused on the development and use of LRRK2 kinase inhibitors (Fig. 1).

After successful inhibition of LRRK2 activity by small molecular inhibitors that resulted in decelerated α-syn aggregation and neurodegeneration in animal models (Daher et al. 2015; Daher et al. 2014), two LRRK2 kinase inhibitors known as DNL201 and DNL151 have been introduced for administration to both healthy volunteers and patients with PD in clinical trials. Safety, tolerability and target engagement of DNL201 has already been established in a phase I, randomized, double-blind, placebo-controlled study in healthy volunteers (Tolosa et al. 2020), as well as in a placebo-controlled, dose-ranging study in 29 PD patients, including subgroups with and without LRRK2 mutation (NCT03710707), according to a press release (Therapeutics 2020). Both doses administered in PD patients were followed by more than 50 % inhibition of LRRK2 and Rab10 phosphorylation in blood, and reduced bis (monoacylglycerol) phosphate in urine, which served as biomarkers of pharmacodynamic measures, and the lower dose was better tolerated. Two ongoing phase I, randomized, double-blind, placebo-controlled trials are ongoing and aim to evaluate safety, tolerability and target engagement of DNL151 in healthy volunteers (NCT04557800) and PD patients (NCT04056689). Other LRRK2 kinase inhibitors that under active development in preclinical models include MLi-2 and PF-06685360 (West 2017).

Another strategy for downregulating LRRK2 activity is the use of anti-sense oligonucleotides (ASOs) to decrease LRRK2 expression levels. This approach carries the advantage of CNS-selective LRRK2 blocking via intraventricular injection of the therapy, avoiding adverse effects of peripheral LRRK2 loss such as alterations in the kidney and lung that have been observed in LRRK2 knockout mice (Herzig et al. 2011). LRRK2-targeted ASOs injected intraventricularly in Gly2019ser mice resulted in decreased α-syn inclusions and loss of nigral dopaminergic neurons, which also had an ameliorating effect on motor deficit (Zhao et al. 2017). Based on these data, a phase I, randomized, triple-blind, placebo-controlled ongoing trial (NCT03976349) will evaluate the safety and tolerability of an intrathecal administration of BIIB094 in patients with PD. As a secondary objective, the study will evaluate the pharmacokinetic profile of BIIB094. The study will include both patients with and without verified presence of pathogenic or likely-pathogenic LRRK2 variants.

Overall, genetic factors associated with PD are distinctly specified and well-studied regarding their function in health and disease, thereby providing more objective tools for patient selection and pharmacodynamic measurements.

### Glucagon‐like peptide 1 receptor agonists and other antidiabetic agents

Biological processes involved in PD share common features with obesity and type 2 diabetes mellitus (T2DM), including the dysregulation of insulin signaling in the brain. The term “brain insulin resistance” has been suggested to describe decreased sensitivity of CNS pathways to insulin, followed by disturbances in synaptic, metabolic and immune response functions (Hoyer 1998). Strategies to normalize insulin sensitivity in neurons have thus been in the spotlight of clinical trials aiming to establish whether they may provide neuroprotective actions.

Glucagon-like peptide-1 (GLP-1) is an endogenous incretin with very short circulating half-life of 1–2 minutes, produced in the enteroendocrine cells of the small intestine (Baggio et al. 2007). GLP-1 receptor agonists (GLP-1 RAs) are agents licensed for the treatment of T2DM. They function through GLP-1 receptor activation, that leads to pancreatic beta-cell proliferation, glucose-dependent insulin secretion, inhibition of glucagon secretion and slowing of gastric emptying (Lovshin et al. 2009). GLP-1 receptors are also found in the brainstem and hypothalamus, and their stimulation is responsible for early satiety (Alvarez et al. 2005). Apart from its glucose lowering effect, GLP-1 receptor stimulation has been investigated in animal models of PD and shown to increase neurogenesis (Bertilsson et al. 2008), to arrest and possible reverse nigrostriatal damage (Harkavyi et al. 2008), and to protect dopaminergic neurons from neurodegeneration (Li et al. 2009). Different mechanisms have been suggested to mediate neuroprotection (Fig. 1 ), including inhibition of microglia activation and matrix metalloproteinase-3 expression (Kim et al. 2009), stimulation of neurogenesis (Bertilsson et al. 2008), and of mitochondrial biogenesis (Fan et al. 2010), and decreased monomeric α-syn load in the striatum (Bassil et al. 2017). In a phase 2 study of the GLP-1 RA exenatide for 12 months in 60 PD patients, the treatment showed efficacy in motor symptoms, and a good safety profile (Athauda et al. 2017). Additional phase 2 trials (Table 2) with semaglutide (NCT03659682), liraglutide (NCT02953665) and lixisenatide (NCT03439943) evaluating treatment effect on motor symptom progression, as well as with exenatide evaluating MRI-based (NCT03456687, Table 2), and FDG-PET based (NCT04305002, Table 2) imaging markers of disease progression are ongoing, as well as a phase 3 trial of exenatide with 200 participants (NCT04232969, Table 2). Also, a phase 2 trial that aims to evaluate the effect of sustained-release formulation of exenatide in symptom improvement in PD, is under way (NCT04269642, Table 2), as well as a dose-ranging trial on the efficacy of pegylated form of exenatide in PD-related motor symptom progression (NCT04154072, Table 2).

The neuroprotective effect of GLP-1 RAs is assumed to be mediated by improved brain insulin sensitivity (Markaki et al. 2020); however, human studies evaluating their biological effect in the CNS are limited. Functional MRI imaging studies have primarily focused on investigating brain networks involved in the anorectic effect of GLP-1 RAs (De Silva et al. 2011; Schlogl et al. 2013), but sparse mechanistic data are available for understanding neuroprotective effects of these drugs. In a more recent trial of exenatide in PD, disease modifying effects measured by nigrostriatal dopamine transporter imaging (DaTscan) were reported (Athauda et al. 2017). Subsequently, brain insulin and Akt signaling pathways were also evaluated in neuronal-derived exosomes and it was shown that exenatide treatment, but not placebo, activated these pathways (Athauda et al. 2019). This significant, secondary analysis of the trial increases understanding of the molecular mechanism underlying the treatment effect and provides a possible biomarker to measure target engagement.

### Calcium targeting therapies

Another potential target for neuroprotection in PD is plasma membrane CAV-1 L-type calcium channel (Ilijic et al. 2011). This concept emerged from the theory of the potentially deleterious consequences of elevated intracellular Ca2+ (Gleichmann et al. 2011) in the vulnerable dopaminergic neurons with strong engagement of CAV-1 L-type Ca2 + channels during autonomous pacemaking (Guzman et al. 2010; Khaliq et al. 2010). Several epidemiologic studies have shown reduced PD risk in patients receiving dihydropyridine calcium channel blockers compared with other antihypertensive agents (Becker et al. 2008; Pasternak et al. 2012; Ritz et al. 2010). Isradipine, a dihydropyridine calcium-channel blocker approved for the treatment of hypertension, has demonstrated a neuroprotective effect (Fig. 1) in animal models of PD (Chan et al. 2007; Guzman et al. 2010; Ilijic et al. 2011), while safety and tolerability of the treatment has been confirmed in clinical trials (Group 2013; Investigators 2020). However, recent results from STEADY-PD III (NCT02168842, Table 2), a 36-month, multicenter, randomized, parallel-group, placebo-controlled trial (Investigators 2020) demonstrated no significant clinical effect of isradipine in progression of early-stage PD, measured as the change of MDS-UPDRS part I to III score in the ON medication state, from baseline to 36 months. Whether the use of higher isradipine dose, or the initiation of treatment with isradipine in even earlier prodromal stages of PD would be more successful is not clear, but better in vivo markers of disease progression and measurement of target engagement are necessary before further studies are undertaken (Maiti et al. 2020).

### Iron targeting therapies

Abnormal iron metabolism is associated with PD and increased intraneuronal iron load has been found in the substantia nigra of patients with PD (Oakley et al. 2007). This increase in iron concentrations can exceed the iron-buffering capacity of complexes, such as neuromelanin and ferritin, and induce neurotoxicity by generating reactive oxygen species (ROS) (Ward et al. 2014). Preclinical studies in animal models of PD demonstrated therapeutic efficacy of iron chelators which could cross the blood-brain barrier, remove excessive intraneuronal iron and reduce ROS formation resulting in increased neuronal survival and normalization of dopamine metabolism (Fig. 1) (Dexter et al. 2011). Results of a phase II, randomized, double-blinded, placebo-controlled, dose-ranging clinical trial on the only available blood-brain-barrier-permeable iron chelator deferiprone, in early-PD patients, showed good efficacy and tolerability. Also, region-specific intraneuronal iron load was assessed by T2* MRI and was shown to decrease in the active-treatment groups, but it did not correlate with significant clinical improvement in terms of changes in the UPDRS scores (Martin-Bastida et al. 2017). Deferipone was also assessed in the FairPARK I trial, which applied delayed-start design and showed that early-start patients had an earlier and more sustainable effect to treatment, both with regard to substantia nigra iron deposit load, and motor symptom severity (Devos et al. 2014). Subsequently, the FairPark II, multi-center trial (NCT02655315, Table 2) was undertaken aiming to evaluate the effect of deferiprone on PD progression measured by the change of total MDS-UPDRS score between baseline and 36 weeks in 372 patients.

### A2A receptor antagonists

In recent years targeting the adenosine A2A receptor has emerged as a promising approach for PD treatment (Cieślak et al. 2008). A2A receptor is a member of the G-protein-coupled receptor family that stimulates adenylate cyclase (Zheng et al. 2019), and is highly expressed in the caudate nucleus, putamen, nucleus accumbens, olfactory tubercles and globus pallidus-pars externa (Mori 2014). Several cellular types, including neurons, astrocytes, oligodendrocytes and microglia express the A2A receptor, which is involved in dopaminergic and glutamatergic neurotransmission (Cervetto et al. 2017), neuroinflammation, and neurodegeneration (Borroto-Escuela et al. 2018; Vuorimaa et al. 2017). In terms of pathophysiological mechanisms of PD, the A2A receptor attracted attention due to its high striatopallidal expression, specifically in GABAergic and glutamatergic striatopallidal medium spiny neurons (Fig. 1) (Shindou et al. 2001; Shindou et al. 2002; Shindou et al. 2003). A2A and dopamine D_2_ receptors form functional heteromeric complexes inducing allosteric inhibition, and A2A receptor activation results in motor inhibition (Beggiato et al. 2016). Furthermore, A2A receptor interacts physically and functionally with glutamate receptors, primarily with the mGlu5 receptor subtype (Beggiato et al. 2016). This interaction facilitates glutamate release in a synergistic manner (Rodrigues et al. 2005), leading to NMDA glutamate receptor activation and an increase in intracellular Ca^2+^ concentrations (Glaser et al. 2020). Thus, inhibition of A2A receptor activity, has been a therapeutic target in many preclinical and clinical studies.

After more than two decades of preclinical and clinical studies, istradefylline is the first non-dopaminergic medication approved by FDA for PD (Chen et al. 2020), proven to significantly reduce OFF-time duration, improve motor outcome, as measured by the UPDRS motor score, as well as to increase duration of time without troublesome dyskinesia (Paton 2020). PET studies were first applied to study A2A receptors in-vivo, in PD patients, showing increased expression in the putamen of patients with dyskinesia compared to controls, and a significant increase in drug-naive patients, after initiation of levodopa treatment (Mishina et al. 2011). Subsequent 11 C-preladenant PET studies in PD patients before and after treatment with istradefylline confirmed sufficient, dose-dependent binding of the drug to A2A receptors in the ventral striatum, caudate and the putamen (Ishibashi et al. 2018). Preladenant is another A2A antagonist which, despite positive results in decreasing OFF time in phase II studies (Factor et al. 2013; Hauser et al. 2011), eventually showed no significant clinical efficacy in following phase III trials, either as monotherapy (Stocchi et al. 2017) or in combination with levodopa-treatment (Hauser et al. 2015). However, the lack of efficacy in the active control group treated with rasagiline raises question on possible issues with study design and conduct (Hauser et al. 2015). Tozadenant showed positive effect on reducing OFF-time duration in a phase IIb, double-blind randomized trial (Hauser et al. 2014), but two subsequent phase III trials (NCT02453386 and NCT03051607) were terminated due to an unexpected emerging safety issue. Vipadenant was investigated in an open-label, PET study and showed occupation of A2A receptors varying from 74 to 94 % in human brain regions, including the putamen, at the lowest daily dose of 2.5 mg, and reached saturation in all regions at 100 mg (Brooks et al. 2010). Nevertheless, no further investigation was performed with regard to clinical efficacy due to safety concerns. KW-6356 is a second-generation A2A antagonist which has shown positive results with regard to motor progression in early-stage PD, as assessed by changes in the MDS-UPDRS motor score from baseline until the end of follow-up, both in combination with levodopa (NCT03703570) and as monotherapy (NCT02939391, Table 2) (Chen et al. 2020).

The approval of istradefylline paves the way for novel therapeutic opportunities of A2A antagonists, while underlining the need for identification of patient subgroups that would benefit from these agents. During the last two decades, genetic studies on the effect coffee consumption have provided important insights in the identification of pharmacogenetic markers that are useful in the prediction of individual responses to caffeine, in PD populations (Amin et al. 2012; Hamza et al. 2011; Popat et al. 2011; Yang et al. 2010). These markers could also be useful to define PD subpopulations that are probable responders to treatment with A2A receptor antagonists and open the way for personalized therapeutic decisions in this field.

### Cell‐replacement therapies

Even though the very first experiments of cell transplantation in animal brains took place in 1890 (Thompson 1890), it was not until late 1970s in Sweden with the development of the 6-hydroxydopamine-lesioned rat model when neural grafting started gaining attention as a potential therapy for PD (Barker et al. 2015). This model enabled selective and irreversible lesioning of the nigrostriatal pathway (Ungerstedt 1968; Ungerstedt et al. 1970; Ungerstedt et al. 1974), opening the way for assessment of the therapeutic potentials of cell transplantation therapies. Based on these preclinical data, an early clinical study showed that solid grafts of adrenal medullary tissue, known to produce catecholamines, including dopamine, placed into the caudate nucleus through an open microsurgical procedure, had significant benefits in two patients with PD (Madrazo et al. 1987). These studies have been followed by several transplantation trials using either mesencephalic dopaminergic neurons (mesDA) obtained from human fetuses (Lindvall et al. 1990) or, alternatively and more promisingly, dopaminergic cells derived from human pluripotent stem cells (hPSCs) (Barker et al. 2017).

Open label studies demonstrated that fetal mesDA neurons transplantation into the striatum of PD patients had potential for clinical improvement as well as graft survival and function, as assessed by clinical measures, neuroimaging (Brundin et al. 2000; Freed et al. 1992; Hagell et al. 1999; Peschanski et al. 1994; Wenning et al. 1997) and post-mortem histological analysis (Hallett et al. 2014; Kordower et al. 1998; Li et al. 2008; Li et al. 2016; Mendez et al. 2005). Two subsequent randomized, double-blind, sham-surgery-controlled clinical trials showed also some clinical improvement, but did not reach the primary end-points (Freed et al. 2001; Olanow et al. 2003). Although some participants demonstrated normalization of DA signaling accompanied by clinical benefits, others showed no improvement or even experienced adverse effects, mainly graft-induced dyskinesias (Barker et al. 2013; Freed et al. 2001; Olanow et al. 2003). Young patients with early-stage PD and no history of dyskinesias prior to the procedure were more probable to benefit from mesDA neural transplantation (Freed et al. 2001; Olanow et al. 2003). These observations were followed by the formation of the European consortium TRANSEURO (NCT01898390) with the principal objective to develop an efficacious and safe treatment for PD patients based on fetal neural grafting. While completion of the study is not expected until later this year, three years after the last transplantation surgery, which occurred at Skåne University Hospital in Lund, Sweden, in early 2018, a recent update has been published, underlining the potential of emerging stem-cell based dopamine-replacement therapies to provide solutions to the previously encountered issues (Barker et al. 2019). The primary outcome measure in TRANSEURO is the change of MDS-UPDRS part 3 score at 36 months post transplantation and change in F-DOPA-PET is included in the secondary end-points.

hESCs and iPSCs were first reported in 1998 and 2007, respectively, and constituted a renewable source of human cells in very primitive developmental stages capable of differentiating in any cell type in the mature human body (Takahashi et al. 2007; Thomson et al. 1998). Major advantages over fetal-derived cells are the availability in near-unlimited numbers, standardization of manufacture procedures, possibility for cryopreservation, as well as cell purity and the potential for more accurate dosing and distribution through microsurgical procedures (Parmar et al. 2020). Based on these advantages, the GForce-PD international corporation was established in 2014 to initiate the first clinical studies of hPSCs transplantation therapies in PD, in Europe, USA, and Japan (Barker et al. 2015).

### Brain connectomic studies and improved precision of neuromodulation targets

The process of altering brain function through direct manipulation of neural activity has long been used to treat patients with neuropsychiatric disorders and deep brain stimulation (DBS) has provided clinical benefit to more than 150 000 patients (Horn et al. 2020) with PD, dystonia and essential tremor (Deuschl et al. 2006; Kupsch et al. 2006; Vitek et al. 2020). Apart from the conventional application in advanced PD, DBS has also been suggested to exert disease-modifying traits (McKinnon et al. 2019). In multiple preclinical studies on rat models, chronic STN electrical stimulation was shown to result in preservation of SNpc dopaminergic neurons (Harnack et al. 2008; Spieles-Engemann et al. 2010; Temel et al. 2006) and an increase of brain-derived neurotrophic factors (Spieles-Engemann et al. 2011) followed by activation of the tropomyosin receptor kinase type B receptor signaling in the nigrostriatal system (Yoshii et al. 2010). Although preclinical experiments suggest potential neuroprotective effects of DBS, results from clinical studies have shown that dopaminergic neuron degeneration remains unaltered (Hilker et al. 2005; Pal et al. 2017), and α-syn burden is not reduced (Pal et al. 2017) in PD patients treated with DBS (Fig. 1).

The development of diffusion MRI and tractography, as well as the increasing prevalence of large databases and computational bioinformatics, has enabled the visualization of this brain-network connectivity and prompted the proposal of “connectivity surgery” that utilizes diffusion tensor imaging (DTI) tractography as targeting modality for DBS-based neural network modulation in movement disorders (Henderson 2012). At the same time, the concept and term of ”circuitopathies” was introduced to describe the disturbances of circuit function and brain-network involved in the pathophysiology of several movement disorders and other neurological diseases (Lozano et al. 2013). Normative connectomics, i.e. atlases of average brain connectivity from large cohorts of participants, were firstly presented in 2017, as a possible predictor of DBS outcome in PD, combining functional and structural connectivity data of open-sourced connectome databases to build a mathematical model that can predict STN-DBS response in patients with PD (Horn et al. 2017). In a recent review, normative connectomics have been compared with patient-specific brain connectivity and have been shown to lead to similar main conclusions about which brain areas are associated with clinical improvement, however patient-specific connectivity profiling was suggested to explain slightly more variance than group connectomes (Wang et al. 2020). Based on these advances, new pathways in neurotherapeutics have opened towards personalized approaches that aim to optimize neuromodulation in a variety of neurological diseases. In a recent, comprehensive overview of DBS technology, the advancements that have contributed to personalized stimulation of specific anatomic structures, real-time record of neural activity and concurrent stimulation adjustments, and identification of key neurocircuitry elements have been highlighted (Krauss et al. 2020). Overview of ongoing DBS trials is beyond the scope of this review.

#### Focused ultrasound as a newly developed neuromodulation technique

Magnetic resonance imaging-guided focused ultrasound (MRgFUS) neurosurgery is emerging as a new option for the treatment of medication-resistant PD. The technique is based on focusing ultrasound beams to specific brain target causing protein denaturation and coagulation necrosis (Harary et al. 2018). Advances in MRI technology have enabled real-time guidance of the procedure by using MR thermometry, facilitating localization of the target below the threshold temperature and ablation of the target above the threshold temperature (Harary et al. 2018). Compared with DBS, MRgFUS neurosurgery appears safe and effective against motor symptoms in PD (Xu et al. 2019), but larger studies with long follow-up are needed to support these results. The first application in PD was performed in 13 medication-resistant patients that received MRgFUS pallidothalamic tractotomy and had a significant UPDRS score reduction at 3 months post intervention, thus indicating feasibility, safety, and accuracy of the method (Magara et al. 2014). In another study, 30 patients with severe PD-related, medication-resistant tremor received MRgFUS and were followed for 1 to 24 months, during which time UPDRS part 2 and PDQ39 scores improved significantly by 6 months, but tremor recurred in 4 patients by 6 to 24 months (Zaaroor et al. 2018). Additional studies in tremor-dominant PD patients (Bond et al. 2017; Fasano et al. 2017; Sperling et al. 2018) and a phase 1 trial in PD dyskinesia showed partly positive results, but were limited by the short follow-up and small patient groups. To date, existing evidence supports the use of MRgFUS to achieve long-term benefits in refractory essential tremor (Meng et al. 2018; Sinai et al. 2019), but further research is required in PD. Several clinical trials (Table 1) are currently ongoing to evaluate the method as a potential therapy in PD symptoms such as dyskinesias and motor fluctuations (NCT02347254, NCT02003248, NCT04002596, NCT03319485, NCT02263885, NCT02246374, NCT03100474). Interestingly, an ongoing study plans to evaluate the application of MRgFUS for the temporary disruption of the blood brain barrier as a potential therapeutic strategy for PD dementia, as well as an effort to overcome problematic pharmaceutical agents’ delivery in their targets of action inside the CNS (NCT03608553, Table 1).

#### Repetitive transcranial magnetic stimulation

Transcranial Magnetic Stimulation (TMS) is a safe and noninvasive technique of electromagnetic brain stimulation (Burke et al. 2019). TMS can probe intracortical circuits and alter cortical activity in the human brain (Fig. 1), where repetitive application has been of therapeutic value in neurological and psychiatric disorders by normalizing aberrant patterns of cortical activity (Burke et al. 2019). Repetitive TMS (rTMS) has been FDA approved for the treatment of mild to moderate medication-resistant depression (Rossi et al. 2009), which has prompted further investigation of its efficacy on a wide range of neuropsychiatric circuitopathies including PD.

rTMS administered over primary frontal cortex at high frequency has been shown to moderately improve motor outcome in PD patients with an average 20 % reduction in the UPDRS motor score (Brys et al. 2016; Khedr et al. 2003; Kim et al. 2015; Yang et al. 2018; Yokoe et al. 2018). In these studies, motor outcome was improved both in the upper and lower extremities independently of the TMS target on hand or foot cortical region, presumably due to plasticity alterations in circuits supplying corticospinal neurons both on proximal and distant brain areas (Underwood et al. 2020). Despite these encouraging results, the need for frequent clinical visits in PD applications has a negative impact on compliance, which can be intensified by mobility issues, as well as the long duration of TMS sessions (Berlim et al. 2014; Yang et al. 2018). Ongoing trials aim to further evaluate the effect of rTMS on PD motor outcome in terms of walking ability as assessed by step variability, step length and gait speed in dual-task walking (NCT04238000, Table 1), and freezing of gait assessed by the change of freezing of gait questionnaire (NCT04431570, Table 1). A phase 2, triple blind, randomized study (NCT04116216, Table 1) aims to investigate whether patients with different PD phenotypes will respond differently to rTMS, as well as to compare the effects of rTMS protocols (high vs. low frequency).

Few studies have investigated the effect of rTMS on levodopa-induced dyskinesias, showing only short-lasting (Brusa et al. 2006; Filipovic et al. 2009; Koch et al. 2005; Sayin et al. 2014; Wagle-Shukla et al. 2007) or no (Flamez et al. 2016) beneficial effect.

Current studies focus on the therapeutic potential of rTMS in PD-associated cognitive dysfunction. A phase 2, randomized controlled trial with quadruple masking including 166 patients with PD mild cognitive impairment (MCI) (NCT03836950, Table 1) will primarily evaluate the effect of rTMS on executive function. Multi-modal neuroimaging will be used in a subgroup of participants to study rTMS-induced neural connectivity changes. Changes in resting state functional connectivity, grey matter volume via voxel-based morphometry and white matter integrity via diffusion tensor imaging between baseline and endpoint will also be assessed. Similarly, in another double blind, randomized trial (NCT02346708, Table 1) 150 patients with PD-MCI will receive bifrontal rTMS and will be assessed by changes in magnetoencephalography connectivity measures as well as clinical cognitive scores. Finally, a ten-center, blinded, sham-controlled, randomized, parallel-group study of fixed-dose, high-frequency and/or low-frequency rTMS in 252 PD patients with depression or cognitive impairment (NCT03552861, Table 1) aims to evaluate the effect of rTMS as an alternative treatment with respect to these common PD symptoms.

## Conclusions

In conclusion, current trends in PD research have moved from dopamine-replenishing, symptomatic therapies to personalized treatments targeted to the restoration of molecular, anatomical and functional integrity of disease-specific brain circuits. Significant technological advances in gene manipulation methods, DBS devices and software, and neuroimaging, in combination with increased awareness of the methodological issues that have so far hampered PD therapeutic research have led to novel pharmacotherapeutic and non-pharmacological strategies that are under ongoing assessment. Additionally, advances in biomarker research and identification of robust, presumably multimodal, markers of pathogenesis and disease progression are of utmost importance for the successful conduct of PD clinical trials aiming to fill the long-lasting deficit in disease modifying, individually tailored treatment options.

## Data Availability

Not applicable.

## Abbreviations

AADC: Aromatic L-amino acid decarboxylase
AAV: Adeno- associated virus
AAV2: Adeno-associated virus serotype 2
ASO: Anti-sense oligonucleotide
CNS: Central nervous system
DaTscan: Dopamine transporter scan
DBS: Deep brain stimulation
DNA: Deoxyribonucleic acid
ER: Endoplasmic reticulum
FDA: Food and drug administration.
^18^F-FDG-PET: ^18^F-fluorodexyglucose positron emission tomography
GAD: Glutamate decarboxylase
GADA: Gamma-aminobutyric acid
GCase: Glucocerebrosidase
GCH1: GTP-cyclohydrolase
GDNF: Glial cell-line derived neurotrophic factor
GLP1: Glucagon-like peptide-1
GSR: Global symptom relief
HDRS: Hamilton Depression Rating Scale
hESCs: Human embryonic stem cells
HLA: Human leucocyte antigen
hPSCs: Human pluripotent stem cells
HSP: Heat shock protein
iPSCs: Induced pluripotent stem cells
LRRK2: Leukine-rich repeat kinase 2
LV: Lentivirus
MADRS: Montgomery-Åsberg Depression Rating Scale
MCI: Mild cognitive impairment
MesDA: mesencephalic dopaminergic
MRgFUS: Magnetic resonance imaging-guided focused ultrasound
MRI: Magnetic resonance imaging
NRTN: Neurturin
PD: Parkinson’s disease
PDQ-39: Parkinson’s disease questionnaire − 39
PET: Positron emission tomography
RA: Receptor agonist
RNA: Ribonucleic acid
ROC: Ras-of-complex
ROS: Reactive oxygen species
rTMS: Repetitive transcranial magnetic stimulation
STN: Subthalamic nucleus
T2DM: Type 2 diabetes mellitus
TH: Tyrosine hydroxylase
TMS: Transcranial magnetic stimulation
UPDRS: Unified Parkinson Disease Rating Scale
α-synuclein: α-syn
